# Editorial: Explainable, trustworthy and responsive intelligent processing of biological resources integrating data, information, knowledge, and wisdom—Volume II

**DOI:** 10.3389/fgene.2022.1114441

**Published:** 2023-01-04

**Authors:** Yucong Duan, Yungang Xu

**Affiliations:** ^1^ College of Computer Science and Technology, Hainan University, Haikou, China; ^2^ School of Basic Medical Science, Xi’an Jiao Tong University, Xi’an, China

**Keywords:** DIKW, DIKW graph, explainability and interpretability, trustworthy AI, responsive ability, knowledge, information retreival, data comprehension

The increasing practice of Artificial Intelligence (AI) in biological and biomedical resources faces challenges of the explainable, trustworthy, responsive AI processing of multi-modal, intertwined, interactive biological and biomedical data, which requires the integration of data, information, knowledge, wisdom and purpose (DIKWP) across objective content and subjective cognition/purpose. Transformations among data, information, knowledge and wisdom open possibilities to comply with uncertainties originating in the incompleteness of data samples, insufficiency of information, vulnerability of invalid knowledge and imbalanced wisdom strategies, towards achieving more precise, robust, reproducibility and less repeated operations of data Research Topic and information synthesis, and more comprehensive knowledge reproducibility through multiple sources reasoning and abstraction. Moreover, alongside the COVID emergency, more and more attention is focused on balancing social welfare, cultural moralities, and the biological practices involving privacy-preserving data Research Topic and legal information usage, under rapid iterations of international political and technical negotiations, towards a responsible AI-enabled AI governance implementing justice, transparency and fairness. This Research Topic aimed to collect the latest research efforts devoted to building capabilities of integration and transformation of multi-modal data, information, knowledge and wisdom in an integrated semantic understanding space unifying subjective purposes and objective formalism, to validate data, retrieve information, abstraction on information to attain knowledge hypotheses, and balanced optimization. In total, nine articles including one review article were published in Frontiers in Genetics.

In the review article Wang et al. proposed a systemic construction towards the mutual incentive among the “social-biological-technological triangle” interaction in hope of interpreting the success and lessons of AI participation in the prevention and treatment of COVID-19.

The Research Topic published eight original research papers that cover a wide range of efforts in applying AI technology in multiple biological and biomedical data sources. Three papers focus on explainable intelligence crossing data graph, information graph and knowledge graph, led by Geng et al., Zhao et al. and Diao et al., respectively. In the article towards addressing the information overloaded problem for personalized recommendation/prescription, Geng et al. proposed a compliment method for integrating subjective sentimental information in the information graph form and objective feature representation in knowledge graph based on representational learning *via* triple-autoencoder. In the article towards leveraging current data intensive or statistical based data graphs into logically explainable knowledge graph in medical industry, Zhao et al. proposed a multi-layers entity extraction architecture to extract object-level entities with “object-attribute” dependencies in the data graph for construction of logic in high-quality medical knowledge graphs based real electronic clinical records. In the article towards constructing an error-avoiding and effort-saving solution in discovering bioinformatics workflow fragments and leveraging historical usages of related activities/services, Diao et al. proposed a workflow Knowledge Graph to unifying common types of data entities and data structural relationship in the data graph of service invoking network, and the implicit information of the information graph in both individual user’s requirements and service communities.

Two article focus on hybrid intelligence resource merging mechanisms crossing incomplete data, inconsistent information and not validated knowledge, led by Wang et al. and Yu and Duan respectively. In the article towards objectifying the knowledge level inconsistency and redundancy originating in the information subjectivity inputted by various biomedical experts, Wang et al. proposed a data-information-knowledge merging approach for biomedical ontology matching *via* a hybrid graph attention network. In the article towards addressing sparsity of data and the cold start of recommendation in prediction of Quality of Services, Yu and Duan proposed a GRU-GAN based learning uniformity over quality data and user characteristic information.

Additionally, three articles presented a trusted resource scheduling method, a miRNA prediction algorithm, and a biological adaptation mechanism, respectively. In the article towards realizing reliable and credible intelligent processing of biological resources, Yu et al. designed a composite service scheduling model under the containers instance mode hybridizing reservation and on-demand. In the article towards understanding miRNAs’ cellular function information and knowledge roles in regulating gene expression, Min et al. proposed to predict essential miRNAs using XGBoost framework with Classification and Regression Trees on various types of sequence-based information features. In the article of towards enhancing the diversity of self-replicating structures, Xu et al. proposed an active self-adaptations in comparison with the passive mechanism through introduction of knowledge rules.

